# Immunogenicity and Predictive Factors Associated with Poor Response after Severe Acute Respiratory Syndrome Coronavirus 2 Vaccination in Lung Transplant Patients

**DOI:** 10.3390/vaccines12070822

**Published:** 2024-07-22

**Authors:** Se Ju Lee, Ala Woo, Jung Ah Lee, Yongseop Lee, Ha Eun Kim, Jin Gu Lee, Song Yee Kim, Moo Suk Park, Su Jin Jeong

**Affiliations:** 1 Division of Infectious Diseases, Department of Internal Medicine, Yonsei University College of Medicine, Seoul 03722, Republic of Korea; playit@inha.ac.kr (S.J.L.); peacefulee@yuhs.ac (J.A.L.); yslee@yuhs.ac (Y.L.); 2 Division of Infectious Diseases, Department of Internal Medicine, Inha University College of Medicine, Incheon 22212, Republic of Korea; 3 Division of Pulmonology and Critical Care Medicine, Department of Internal Medicine, Yonsei University College of Medicine, Seoul 03722, Republic of Korea; alwoo@yuhs.ac (A.W.); dobie@yuhs.ac (S.Y.K.); pms70@yuhs.ac (M.S.P.); 4 Department of Thoracic and Cardiovascular Surgery, Yonsei University College of Medicine, Seoul 03722, Republic of Korea; gracehn@yuhs.ac (H.E.K.); csjglee@yuhs.ac (J.G.L.)

**Keywords:** COVID-19, lung transplantation, vaccination

## Abstract

Lung transplant patients are more likely to develop severe coronavirus disease 2019 (COVID-19) compared with the general population and should be vaccinated against severe acute respiratory syndrome coronavirus 2 (SARS-CoV-2). However, previous studies have reported reduced vaccination immunogenicity in lung transplantation patients. We aimed to investigate the serological response and associated factors after SARS-CoV-2 vaccination in this population. Lung transplant patients without a history of contracting coronavirus disease who had received a second or higher dose of SARS-CoV-2 vaccination were enrolled. The anti-SARS-Cov-2 spike and neutralizing antibody levels were measured in blood samples. Firth’s logistic regression analysis was performed to assess the factors associated with non-response after vaccination. Forty-six lung transplant patients were enrolled, of which sixteen (34.8%) showed a serological response to vaccination. All patients who received anti-SARS-CoV-2 vaccination before transplantation (*n* = 5) exhibited a serological response. No significant difference was observed in anti-SARS-CoV-2 S antibody or neutralization titers based on the number and timing of vaccination. Firth’s logistic regression showed an association between lower hemoglobin levels (odds ratio, 0.59; confidence interval, 0.35–0.92; *p* = 0.017) and non-response to SARS-CoV-2 vaccination. Lung transplant patients showed poor serologic responses after SARS-CoV-2 vaccination in this pilot study; anemia may be associated with this poor response.

## 1. Introduction

Although the severity has decreased since the omicron variant became the dominant variant of concern, severe acute respiratory syndrome coronavirus 2 (SARS-CoV-2) can still cause severe illness in patients undergoing solid organ transplantation (SOT) [1,2]. SOT patients are more likely to develop severe coronavirus disease 2019 (COVID-19) than the general population, and the hospitalization rate due to COVID-19 is reported to be 26–63%, with a mortality rate of 13–30% [3]. Among SOT patients, lung transplant patients have a poorer prognosis for COVID-19 [4]. Additionally, the established relationship between respiratory infections and chronic lung allograft dysfunction makes SARS-CoV-2 a significant threat to lung transplant patients [5].

Vaccination is effective in preventing infection and severe disease; therefore, SARS-CoV-2 vaccination is recommended for SOT patients and those on the waitlist [6]. Currently, the Centers for Disease Control and Prevention recommends that unvaccinated SOT patients recieve two or three doses of the same brand of updated SARS-CoV-2 vaccine [7]. However, the immunogenicity of the SARS-CoV-2 vaccine in SOT patients is lower than that in the general population [3]. SOT patients showed decreased response to vaccination due to the use of immunosuppressants to prevent graft rejection [8]. Previous studies reported pooled response rates of 43.6% after the second dose and 55.1% after the third dose in SOT patients. Moreover, previous studies have reported reduced vaccination immunogenicity in lung transplant patients compared with other SOT or immunocompromised patients [3,9,10]. Ethnicity may also affect immunogenicity, and studies on the immunogenicity of lung transplant patients in the Republic of Korea are still lacking [11,12]. Hence, this study aimed to investigate the serological responses and associated factors after SARS-CoV-2 vaccination in lung transplant patients.

## 2. Materials and Methods

The study participants comprised lung transplant recipients who underwent lung transplantation and received follow-up care at Severance Hospital, a tertiary hospital in the Republic of Korea. This study was approved by the Institutional Review Board of the Yonsei University Health System Clinical Trial Centre (4-2021-0554). Written informed consent was obtained from all participants. This study was conducted in accordance with the ethical standards of the 1964 Declaration of Helsinki and its subsequent amendments. Lung transplant patients who received a second or higher dose of SARS-CoV-2 vaccination were included in the study. Meanwhile, patients (1) with previously confirmed COVID-19 and (2) who tested positive for SARS-CoV-2 nucleocapsid antibody were excluded. Blood samples were collected on the day of enrollment if >14 days had passed since the last vaccination; otherwise, samples were collected 14 days after the last vaccination.

All relevant clinical and laboratory data were collected from the electronic medical records. Laboratory tests were collected based on sampling dates. Immunosuppressant use was recorded from the first vaccination until the sampling date.

### 2.1. Anti-SARS-CoV-2 Antibody Assay

Anti-SARS-Cov-2 antibodies were measured in the blood samples of vaccinated lung transplant patients. Measurements were performed using a quantitative Elecsys Anti-SARS-CoV-2 S assay (Roche Diagnostics, Mannheim, Germany) with a range of 0.40 of 250 U/mL. Samples with values >250 U/mL were diluted to the linear range of the assay (1:10–2500 U/mL and 1:100–25,000 U/mL). A value of ≥0.8 U/mL indicated a positive result.

Anti-nucleocapsid antibodies were recognized as markers of a previous occurrence of COVID-19. Elecsys Anti-SARS-CoV-2 N assay was used to exclude participants with a COVID-19 history. A cutoff index of ≥1.0 indicated a positive result.

### 2.2. Neutralization Assay

The SARS-CoV-2 wild-type virus (NCCP43326) and omicron strain (NCCP43408) were used in the plaque reduction neutralization test (PRNT). Neutralization assays were performed using Vero E6 cells. The serum samples were inactivated and sequentially diluted two-fold (starting from 1:40), and the virus, diluted to a concentration of 3 × 10^3^ plaque-forming units (PFU)/mL, was equally distributed into the diluted serum at a concentration of 50 PFU (30–60 plaques observed). The 50% neutralization dilution (ND_50_) was expressed as the reciprocal dilution of serum that showed reduced plaque number by 50% compared with that in the positive control. The ND_50_ titers were calculated using the Spearman–Kärber method. 

### 2.3. Statistical Analysis

The study population was classified according to their serological response to SARS-CoV-2 vaccination. The differences in patient characteristics and outcomes between the groups were analyzed using the chi-square test or Fisher’s exact test for categorical variables, and the *t*-test or Wilcoxon rank-sum test for continuous variables. The distribution of continuous variables was assessed using the Shapiro–Wilk test. The Kruskal–Wallis test was used to compare continuous variables according to the number of vaccinations received. The results of quantitative plasma cytomegalovirus polymerase chain reaction below the detectable range were treated as zero for statistical analysis. The neutralization titers were analyzed and expressed as geometric mean titers (GMTs) along with the corresponding 95% confidence intervals (CI). Pearson’s correlation analysis was performed to investigate the relationship between the anti-SARS-CoV-2 S antibody titer and other variables. Firth’s logistic regression analysis was performed to assess the factors associated with non-response to SARS-CoV-2 vaccination. Variables with a *p* value of <0.1 in the univariate analyses, and deemed clinically relevant, were included in the multivariable model. Statistical significance was set at a *p* value of <0.05. All statistical analyses were performed using the R V.4.0.5 (The R Foundation for Statistical Computing, Vienna, Austria). 

## 3. Results

By May 2022, 445 patients had undergone lung transplantation at Severance Hospital and received follow-up care. Of them, 46 were enrolled in the study. Thirty patients (65.2%) were men, and the median age was 59 years (interquartile range (IQR): 46–66 years). A total of 5 patients received their first SARS-CoV-2 vaccine dose before transplantation. Further, 9 patients (19.6%) received two doses, 31 patients (67.4%) received three doses, and 6 patients (13.0%) received four doses of vaccination. Among these patients, 2 (4.3%) received adenoviral vector vaccines alone, 22 (47.8%) received mRNA vaccines alone, and 22 (47.8%) received heterogeneous vaccinations.

In the study population, 16 patients (34.8%) showed a serological response to vaccination. All patients who received the first vaccine dose before lung transplantation exhibited a serological response. Among 41 patients who received SARS-CoV-2 vaccination after lung transplantation, the responder group (11 patients, 26.8%) showed significantly higher hemoglobin levels (13.5 g/dL, IQR: 12.8–15.0 and 11.8 g/dL, IQR: 10.5–13.4, *p* = 0.01) and lower C-reactive protein levels (4 mg/dL, 2.5–6.0 and 9.0 mg/dL, 6.0–21.0, *p* = 0.015) (Table 1). Among the SARS-CoV-2 vaccination non-responders, four patients were diagnosed with COVID-19 by May 2022, and one patient required oxygen therapy.

Firth’s logistic regression showed that lower hemoglobin levels (odds ratio, 0.59; confidence interval, 0.35–0.92; *p* = 0.017) were associated with non-response to SARS-CoV-2 vaccination after lung transplant (Table 2). The number or type of vaccinations did not show a significant association with the response to SARS-CoV-2 vaccination.

**Figure 1:**
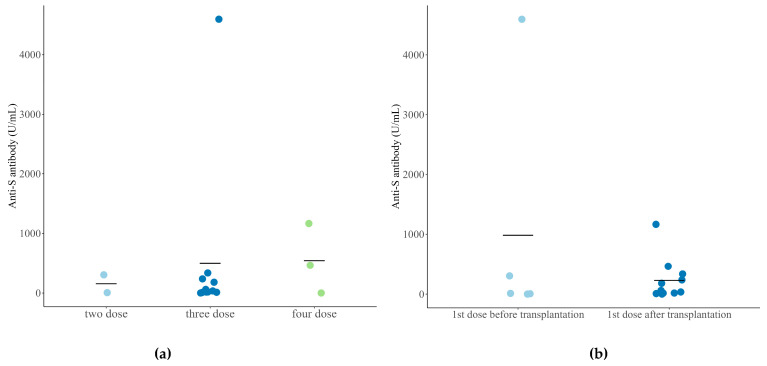
Anti-SARS-CoV-2 S antibody titer according to the number of vaccination (**a**) and start of vaccination before/after transplantation (**b**).

Figure 1 shows the anti-SARS-CoV-2 S antibody titer according to the number of vaccinations and the timing of vaccination (before or after transplantation). No significant difference was observed between the two groups (Kruskal–Wallis test, *p* = 0.814; Wilcoxon rank sum test, *p* = 0.583). In the neutralization assay, the PRNT ND_50_ values for the wild-type virus and the omicron variant did not differ depending on the number of vaccinations (*p* = 0.374, *p* = 0.941) (Supplementary Figure S1). In addition, the PRNT ND_50_ values for the wild-type virus and omicron variant were not significantly different according to the timing of the first SARS-CoV-2 vaccination dose (*p* = 0.820, *p* = 0.913) (Supplementary Figure S2). No significant difference was also found between the neutralization titers of the wild-type virus and omicron variant (*p* = 0.736). Conversely, the correlation plot showed a positive correlation between lymphocyte count and anti-SARS-CoV-2 S antibody titer (*r* = 0.56, *p* = 0.076).

## 4. Discussion

This study analyzed the serological response and factors associated with response to SARS-CoV-2 vaccination in lung transplant patients. Only 34.8% of lung transplant patients and 26.8% of those patients who received the first vaccination dose before transplantation showed a serological response to the SARS-CoV-2 vaccine, consistent with the results of previous studies [3,9]. Moreover, the anti-spike antibody and neutralization titers in SOT patients are lower than those in the general population [10,13,14]. Among SOT patients, lung transplant patients are at greater risk of contracting COVID-19; therefore, further measures to strengthen the serologic response and neutralization capacity are essential.

Patients with SOTs often exhibit inadequate humoral response to SARS-CoV-2 vaccination even after receiving the third dose of vaccination, which is considered a primary series for immunocompromised patients; therefore, studies exploring booster doses have indicated varying efficacy [15,16,17,18]. Meanwhile, several studies have suggested that a fourth dose of vaccination can cause an increase in the antibody response and neutralizing capacity. However, another study suggested that the neutralizing capacity against the omicron variant was insufficient even after the fourth dose [17]. In our study, despite a notable number of patients receiving a fourth dose, no significant increase was noted in the anti-SARS-CoV-2 S antibody titer, neutralizing titer for wild-type virus, or neutralizing titer for the omicron variant compared with the third dose vaccination group. A few studies have suggested that the fifth dose of vaccination improves the humoral response in SOT patients. However, further investigation is needed on the effectiveness of the booster dose for lung transplant patients due to their underrepresented proportions in current studies [18,19].

Generally, potential SOT recipients are advised to receive SARS-CoV-2 vaccination before transplantation [20,21]. Hamaya et al. demonstrated a high seropositivity rate in kidney transplant recipients who were vaccinated against SARS-CoV-2 before kidney transplantation. Similarly, our study showed a serological response in all five patients who received vaccination prior to lung transplantation [22]. However, our study showed no significant difference in the anti-SARS-CoV-2 S antibody titer or neutralization titer compared with recipients vaccinated post-transplantation. Of the five patients, two underwent lung transplantation after the first vaccine dose, two underwent transplantation after the second dose, and one underwent transplantation after the third dose. Moreover, two patients who underwent lung transplantation after the second dose did not receive a third dose after transplantation and participated in this study. In patients with SOT, the third dose of vaccination is considered the primary series; therefore, incomplete dosing could impact serologic responses in these patients. Additionally, two patients vaccinated pre-transplantation received subsequent doses two months post-transplantation. Gleeson et al. reported that booster vaccination during the early post-transplantation period did not yield sufficient response in patients vaccinated pre-transplantation [23]. Therefore, early vaccination after transplantation may have affected the results of this study.

Anemia is prevalent among lung transplant patients, with various proposed mechanisms for post-lung transplantation anemia [24,25]. Iron deficiency anemia, a primary cause of anemia, has been studied extensively, with findings suggesting that it does not significantly affect the immune response after SARS-CoV-2 vaccination [26,27]. However, several studies on dialysis patients indicated a potential link between anemia and poor serological responses after vaccination [28,29]. Anemia in lung transplant patients may signify underlying nutritional abnormalities or chronic inflammation, both of which could contribute to impaired vaccination responses [30]. Further research is needed to determine the association between anemia and serological responses after vaccination in lung transplant patients.

This study has several limitations. First, the participants were enrolled solely from a single institution, which may not fully represent the broader population of lung transplant patients. Second, the small sample size could restrict our ability to conclusively identify factors influencing serological responses, including variations in vaccination protocols and types of vaccines administered. Third, the interval between vaccination and analysis of humoral responses varied among participants in this study. A more consistent timeframe from vaccination to sampling would have allowed for a more precise comparison of humoral immunity. Fourth, we could not analyze cell-mediated immunity, and analyzing only the serologic response may have led to underestimation of the immunogenicity of SARS-CoV-2 vaccination in lung transplant patients. Fifth, SARS-CoV-2 vaccines used during our study period are now updated. Currently, the Omicron XBB.1 monovalent vaccine is used, and one dose of the XBB.1 monovalent vaccine is recommended regardless of previous vaccination history in South Korea. Further research on the updated vaccines is needed.

## 5. Conclusions

In conclusion, lung transplant patients showed poor serological responses after SARS-CoV-2 vaccination in this pilot study, and anemia may be associated with a poor serological response. Further studies are needed to confirm the results of this pilot study.

## Figures and Tables

**Table 1:** Characteristics of vaccine responders and non-responders among lung transplant patients vaccinated after transplantation.

	Responders (*n* = 11)	Non-Responders (*n* = 30)	*p*-Value
Age, y	56.0 (42.5–66.0)	60.0 (48.0–64.0)	0.768
Male Sex	7 (63.6%)	18 (60.0%)	>0.99
BMI, kg/m^2^	22.1 (21.3–22.7)	22.1 (20.0–23.6)	0.837
Number of vaccinations			0.195
2nd dose	0	7 (23.3%)	
3rd dose	9 (81.8%)	20 (66.7%)	
4th dose	2 (18.2%)	3 (10.0%)	
Vaccination type			0.711
Viral vector vaccine only	0	1 (3.3%)	
AZD1222	0	1 (3.3%)	
mRNA vaccine only	5 (45.5%)	16 (53.3%)	
BNT162b2	4 (36.4%)	14 (46.7%)	
mRNA-1273	1 (9.1%)	2 (6.7%)	
Heterogenous vaccination	6 (54.5%)	13 (43.3%)	
AZD1222–BNT162b2	5 (45.5%)	9 (30.0%)	
AZD1222–mRNA-1273	1 (9.1%)	4 (13.3%)	
Post-transplant days	1647.0 (884.0–1928.0)	1259.5 (907.0–2229.0)	0.942
Transplantation–last vaccination interval, days	1549.0 (771.0–1888.0)	1159.5 (819.0–2175.0)	0.965
Transplantation–first vaccination interval, days	1348.0 (638.5–1662.5)	1034.5 (632.0–1918.0)	0.965
Last vaccination–sampling interval, days	119.0 (81.0–148.0)	99.5 (48.0–130.0)	0.331
Comorbidity			
Hypertension	3 (27.3%)	16 (53.3%)	0.259
Diabetes mellitus	6 (54.5%)	13 (43.3%)	0.776
Coronary artery disease	1 (9.1%)	8 (26.7%)	0.436
Heart failure	0	1 (3.3%)	>0.99
Chronic kidney disease	4 (36.4%)	14 (46.7%)	0.815
Chronic liver disease	2 (18.2%)	4 (13.3%)	>0.99
Connective tissue disease	3 (27.3%)	9 (30.0%)	>0.99
Solid cancer history	1 (9.1%)	2 (6.7%)	>0.99
Hematologic malignancy history	0	6 (20.0%)	0.268
Immunosuppressant			0.444
Steroid, Mycophenolate mofetil, Cyclosporine	0	2 (6.7%)	
Steroid, Mycophenolate mofetil, Tacrolimus	11 (100.0%)	26 (86.7%)	
Steroid, Tacrolimus	0	2 (6.7%)	
Steroid dose (Prednisolone equivalent), mg	6.0 (5.0–7.5)	7.5 (5.0–10.0)	0.322
Basiliximab use during induction therapy	3 (27.3%)	13 (43.3%)	0.567
ATG use during induction therapy	0	1 (3.3%)	>0.99
Laboratory data			
White blood cell count, 10^3^/μL	5.9 (5.0–6.9)	6.3 (5.0–7.9)	0.612
Lymphocyte count, 10^3^/μL	1.4 (1.1–1.8)	1.3 (1.0–1.8)	0.988
Hemoglobin, g/dL	13.5 (12.8–15.0)	11.8 (10.5–13.4)	0.010
Platelet, 10^3^/μL	249.0 (212.0–273.0)	208.0 (188.0–260.0)	0.410
Blood urea nitrogen, mg/dL	22.5 (14.9–25.0)	26.5 (20.5–36.6)	0.070
Creatinine, mg/dL	1.1 (0.9–1.3)	1.0 (0.9–1.5)	0.724
eGFR (CKD-EPI)	68.0 (61.0–86.5)	65.0 (44.0–89.0)	0.324
C-reactive protein, mg/dL	4.0 (2.5–6.0)	9.0 (6.0–21.0)	0.015
Quantitative Plasma Cytomegalovirus PCR *, IU/mL *	0.0 (0.0–0.0)	0.0 (0.0–0.0)	0.804

* The results of quantitative plasma cytomegalovirus polymerase chain reactions below the detectable range were treated as zero for statistical analysis. BMI, body mass index; ATG, anti-thymocyte globulin; GFR, glomerular filtration rate; PCR, polymerase chain reaction.

**Table 2:** Univariable and multivariable analyses for SARS-CoV-2 vaccine non-response among lung transplant patients.

	Univariate Analysis				Multivariate Analysis			
	OR	2.5%	97.5%	*p*-Value	OR	2.5%	97.5%	*p*-Value
Hemoglobin per 1 g/dL	0.58	0.34	0.88	0.009	0.59	0.35	0.92	0.017
Vaccination with more than two doses	0.14	0.00	1.29	0.091	0.17	0.00	1.92	0.175
Blood urea nitrogen	1.05	0.99	1.15	0.117				
C-reactive protein	1.00	0.99	8.11	0.542				
Age	1.01	0.96	1.06	0.666				
Male sex	0.89	0.21	3.45	0.865				
Vaccine type (ref: adenovirus vector vaccine)								
mRNA vaccine	1.00	0.01	21.87	>0.99				
Heterogenous vaccine	0.69	0.00	14.97	0.825				

## Data Availability

The data presented in this study are available from the corresponding author on reasonable request.

## Supplementary Materials

The following supporting information can be downloaded at: https://www.mdpi.com/article/10.3390/vaccines12070822/s1, Figure S1. Neutralization titer against wild-type virus (A) and Omicron variant (B) according to the number of vaccination. Figure S2. Neutralization titer against wild-type virus (A) and Omicron variant (B) according to start of vaccination before/after transplantation. Figure S3. Correlation plot of Anti-SARS-CoV-2 S antibody titer according and lymphocyte counts.
