# Supplementary material for: Immunogenicity and Predictive Factors Associated with Poor Response after Severe Acute Respiratory Syndrome Coronavirus 2 Vaccination in Lung Transplant Patients
Source: Vaccines (Basel). 2024 Jul 22;12(7):822. doi: 10.3390/vaccines12070822 (PMC11281714; doi:10.3390/vaccines12070822)
Supplement: Supplementary file 1 [file vaccines-12-00822-s001.zip › vaccines-3079204-supplementary.pdf]

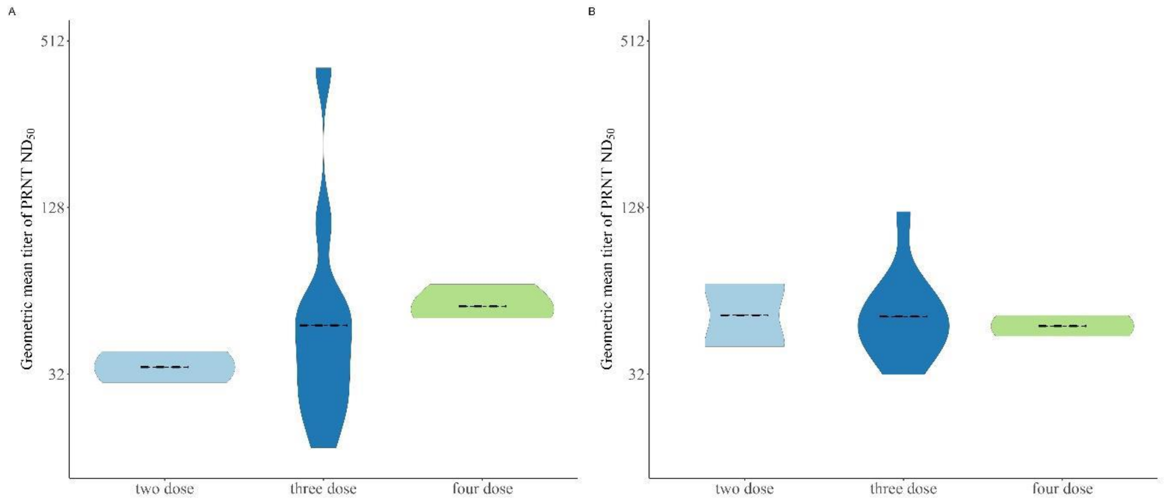

Figure S1. Neutralization titer against wild-type virus (**A**) and Omicron variant (**B**) according to the number of vaccination.

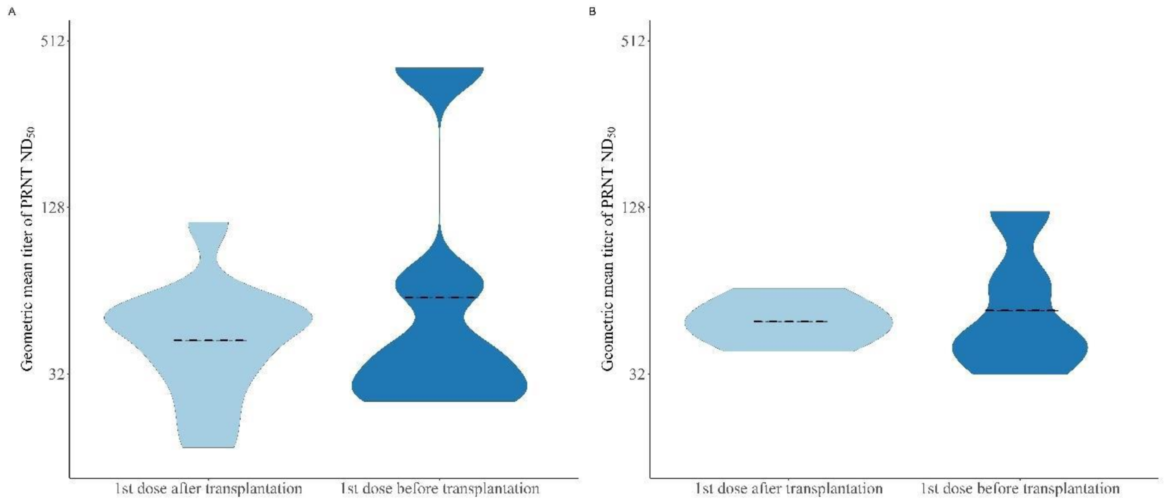

Figure S2. Neutralization titer against wild-type virus (**A**) and Omicron variant (**B**) according to start of vaccination before/after transplantation.

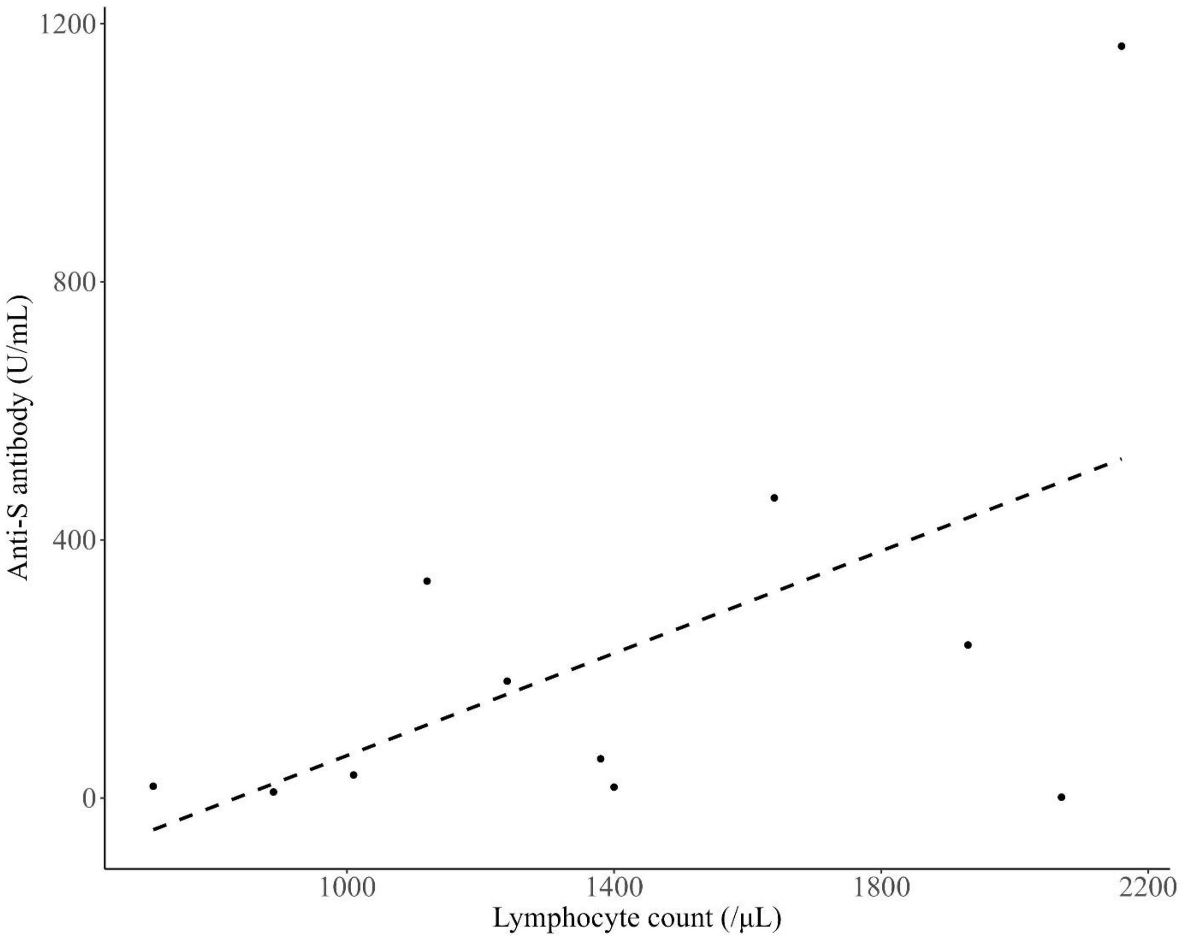

Figure S3. Correlation plot of Anti-SARS-CoV-2 S antibody titer according and lymphocyte counts.
